# Protective Effect of Chokeberry (*Aronia melanocarpa* L.) Extract against Cadmium Impact on the Biomechanical Properties of the Femur: A Study in a Rat Model of Low and Moderate Lifetime Women Exposure to This Heavy Metal

**DOI:** 10.3390/nu9060543

**Published:** 2017-05-25

**Authors:** Małgorzata M. Brzóska, Alicja Roszczenko, Joanna Rogalska, Małgorzata Gałażyn-Sidorczuk, Magdalena Mężyńska

**Affiliations:** Department of Toxicology, Medical University of Bialystok, Adama Mickiewicza 2C street, 15-222 Bialystok, Poland; alicja.roszczenko@umb.edu.pl (A.R.); joanna.rogalska@umb.edu.pl (J.R.); malgorzata.galazyn-sidorczuk@umb.edu.pl (M.G.-S.); mmezynska1@student.umb.edu.pl (M.M.)

**Keywords:** *Aronia melanocarpa* berries, bone biomechanical properties, cadmium, chokeberries, female rats, polyphenols, procollagen, protection

## Abstract

The hypothesis that the consumption of *Aronia melanocarpa* berries (chokeberries) extract, recently reported by us to improve bone metabolism in female rats at low-level and moderate chronic exposure to cadmium (1 and 5 mg Cd/kg diet for up to 24 months), may increase the bone resistance to fracture was investigated. Biomechanical properties of the neck (bending test with vertical head loading) and diaphysis (three-point bending test) of the femur of rats administered 0.1% aqueous chokeberry extract (65.74% of polyphenols) or/and Cd in the diet (1 and 5 mg Cd/kg) for 3, 10, 17, and 24 months were evaluated. Moreover, procollagen I was assayed in the bone tissue. The low-level and moderate exposure to Cd decreased the procollagen I concentration in the bone tissue and weakened the biomechanical properties of the femoral neck and diaphysis. Chokeberry extract administration under the exposure to Cd improved the bone collagen biosynthesis and femur biomechanical properties. The results allow for the conclusion that the consumption of chokeberry products under exposure to Cd may improve the bone biomechanical properties and protect from fracture. This study provides support for *Aronia melanocarpa* berries being a promising natural agent for skeletal protection under low-level and moderate chronic exposure to Cd.

## 1. Introduction

The growing occurrence of osteoporosis with bone fracture in inhabitants of industrialized countries, which has generated an increasing amount of attention in recent years, has been focused on environmental risk factors for bone damage [1,2,3] and numerous efforts have been undertaken to find effective ways of protecting bone [4,5,6]. Among the factors that may be useful in this protection, a subject of special interest is dietary products that are rich in biologically active substances, characterized by a well-defined beneficial impact on bone metabolism, including polyphenolic compounds that occur in green tea and some fruit and vegetables [4,5,6,7].

More numerous epidemiological data provide evidence that important environmental risk factors for the increasing incidence of osteoporosis are toxic heavy metals, including cadmium (Cd) [1,2,3,8,9,10]. Due to the wide distribution of this metal in the environment and food pollution, as well as its presence in tobacco smoke, the whole population is exposed to this metal during their lifetime [8,9,10,11,12]. Bone damage is one of the main unfavourable health effects of long-term exposure to this xenobiotic in both human [1,8,9,10,11,12] and experimental animals [13,14,15,16,17,18,19]. We have reported, for a rat model with environmental human exposure to Cd, that this metal disturbs bone metabolism and weakens the biomechanical properties of long bones and lumbar spine vertebral bodies, which may even result in femoral neck and vertebral fractures [13,14,15,16,17,18]. In recent years, numerous epidemiological data have shown that even low-level lifetime exposure to this heavy metal may decrease the bone mineral density (BMD) and contribute to osteoporosis with an increased risk of bone fracture in the general population [1,8,9,10]. Moreover, the forecasts indicate that the exposure of the general population to this xenobiotic will increase in the future decades [11]. Thus, it seems very important to recognize effective ways of preventing these health effects due to exposure to Cd, including its impact on the skeleton.

The attention of researchers regarding effective agents that may play a role in protecting against the effects of Cd action has been focused on natural products, including those rich in polyphenolic compounds [20,21,22]. Due to the multidirectional favourable action of polyphenols (antioxidative, anti-inflammatory, anticoagulative, antiatherogenic, antidiabetic, antibacterial, and anticancer), products abundant in these compounds are recommended to be used as functional food in the case of cardiovascular diseases, diabetes, neurodegenerative disorders, urinary tract infection, non-alcoholic fatty liver disease, and chemotherapy [20,22,23]. Moreover, polyphenols are also known for their beneficial impact on the skeleton [4,6,7,24].

Available data, including our own findings, show that some plant products rich in polyphenolic compounds, including the berries of *Aronia melanocarpa* (chokeberries; [Michx.] Elliott, Rosaceae), possess the potential to protect from various unfavourable effects that result from the exposure to Cd [21,22,25,26,27]. Recently, using a rat model of lifetime low-level and moderate female exposure to Cd (1 and 5 mg Cd/kg diet), we have revealed that the consumption of an extract from the berries of *A. melanocarpa* (AE) protected against these heavy metal-induced disturbances in the bone turnover and bone mineral status (findings are summarized as Tables S1 and S2) [26,27]. Because the mineral status of the bone tissue determines its biomechanical properties and susceptibility to fracture [28], taking into account our recent findings [25,26,27], we have hypothesized that the consumption of AE under Cd exposure may also improve the bone biomechanical properties and, in this way, decrease the bone susceptibility to fracture. The aim of the present study was to investigate this hypothesis. For this purpose, the impact of AE administration under chronic low-level and moderate exposure to Cd on the biomechanical properties of the femoral neck and femoral diaphysis was estimated for the animals in which we have revealed the protective impact of the extract against the body burden of Cd and disorders in the bone tissue metabolism [25,26,27]. Moreover, because the bone biomechanical properties are determined not only by the bone mineral status, but also by the bone matrix composed mainly of collagen [29,30], the concentration of procollagen I (PC I) in the bone tissue, as a marker of collagen biosynthesis, was assayed.

## 2. Materials and Methods

### 2.1. Animals

One hundred and ninety-two young (aged three to four week) female Wistar rats [Crl: WI (Han)] purchased from the certified Laboratory Animal House (Brwinów, Poland) were used. The animals were housed in controlled conventional conditions (temperature 22 ± 2 °C, relative humidity 50 ± 10%, 12-h light/dark cycle) and had free access to drinking water and feed (Labofeed B diet through the first three months and then a Labofeed H diet; Label Food ‘Morawski’, Kcynia, Poland) [25,26].

### 2.2. Cd Diets

The diets containing 1 and 5 mg Cd/kg were prepared by the addition, at the stage of production, of appropriate amounts of CdCl_2_ × H_2_O (POCh, Gliwice, Poland) into the ingredients of the standard feed (Labofeed B and Labofeed H diets). The mean Cd concentration determined by us (by atomic absorption spectrometry method) in the diets reached 1.09 ± 0.11 mg/kg and 4.92 ± 0.78 mg/kg (mean ± SE), respectively, whereas its mean concentration in the standard Labofeed diets was 0.0584 ± 0.0046 mg/kg.

### 2.3. A. melanocarpa Extract

Powdered aronia extract was received from Adamed Consumer Healthcare (Tuszyn, Poland). According to the producer, the extract contained 65.74% of polyphenols (including 18.65% of anthocyanins). Although the polyphenols content in the powdered *A. melanocarpa* extract was certified and is widely reported [22,23,31,32], the phytochemical profile of the extract was evaluated and the concentrations of total polyphenols, phenolic acids (including chlorogenic acid), flavonoids, proanthocyanidins, and anthocyanins (including cyanidin 3-*O*-β-galactoside, cyanidin 3-*O*-α-arabinoside, and cyanidin 3-*O*-β-glucoside) were quantified by us [26] (Table S3). According to the producer’s declaration and literature data [22,31], the extract also contained other components such as pectins, sugar, sugar alcohols (sorbitol, parasorboside), triterpenes, carotenoids, and phytosterols, as well as minerals and vitamins.

### 2.4. Study Design

The study was approved by the Local Ethics Committee for Animal Experiments in Bialystok (Poland) and was performed following the ethical principles, institutional guidelines, and international Guide for the Use of Animals in Biomedical Research.

The rats were allowed to adjust to the experimental facility for five days before the study was started and were randomly divided into six groups, each containing 32 animals. One group received 0.1% aqueous solution of aronia extract alone as the only drinking fluid (AE group), two groups were intoxicated with Cd alone via the diets containing 1 and 5 mg Cd/kg (Cd_1_ group and Cd_5_ group), and the next two groups received the AE during the whole course of the exposure to Cd (Cd_1_ + AE group and Cd_5_ + AE group) for 3, 10, 17, and 24 months. The Cd_1_ group and Cd_5_ group, unlike the Cd_1_ + AE group and Cd_5_ + AE group, did not receive the AE. The last group, maintained on redistilled water (containing < 0.05 μg Cd/L and not completely deprived of bioelements necessary for the bone health) and standard Labofeed diet, served as a control.

The daily intake of Cd throughout the 24-month exposure to the 1 and 5 mg Cd/kg diets, irrespective of whether this xenobiotic was administered alone or in conjunction with the AE, reached 37.50–84.88 μg/kg b.w. and 196.69–404.76 μg/kg b.w., respectively. Cd intake in the control group and AE group was negligible compared to its intake in the Cd_1_, Cd_1_ + AE, Cd_5_, and Cd_5_ + AE groups (Table S4) [25,26].

The 0.1% AE was prepared daily by dissolving 1 g of the powdered aronia extract in 1 L of redistilled water. The total polyphenols concentration in the 0.1% aqueous AE reached 0.612 ± 0.003 mg/mL (mean ± SE) and was stable for 24 h after this solution preparation, while the Cd concentration was <0.05 μg/L [26]. The daily intake of polyphenolic compounds during the whole experiment, irrespective of the manner of their administration, reached 41.5–104.6 mg/kg b.w. and did not differ, regardless of whether the AE was administered alone or under the treatment with Cd (Table S5) [26].

At the end of the 3rd, 10th, 17th, and 24th month of the experiment, eight rats of each group (except for seven animals in the AE group, and the groups treated with 1 and 5 mg Cd/kg diet alone after 24 months), after overnight starvation, were subjected to anaesthesia with Morbital (pentobarbital sodium and pentobarbital 5:1, 30 mg/kg b.w., *i*.*p.*). The whole blood was taken by cardiac puncture with and without anticoagulant (heparin), and various organs and tissues, including the right femur used in the present study, were dissected.

The femur was immediately cleaned of all adherent soft tissues and weighed with an automatic balance (OHAUS, Switzerland, accuracy to 0.0001 g). Moreover, the bone length and the anterior–posterior (A–P) and medial–lateral (M–L) diameters at the midpoint of the diaphysis were measured with an electronic calliper (±0.02 mm). All the measurements were performed by the same investigator. The precision of these measurements (determined by three measurements of three bones), expressed as a coefficient of variation (CV) for the femur length and diameter, was <0.45% and 0.5%, respectively. The femur of each rat was stored in physiological saline (0.9% sodium chloride) at −20 °C until biomechanical testing was performed. After biomechanical testing, the bone slices collected from the distal epiphysis and diaphysis of the femur were subjected to a PC I measurement.

The experimental model has been described in detail in our previous reports [25,26,27].

### 2.5. Bone Biomechanical Testing

**Figure 1:**
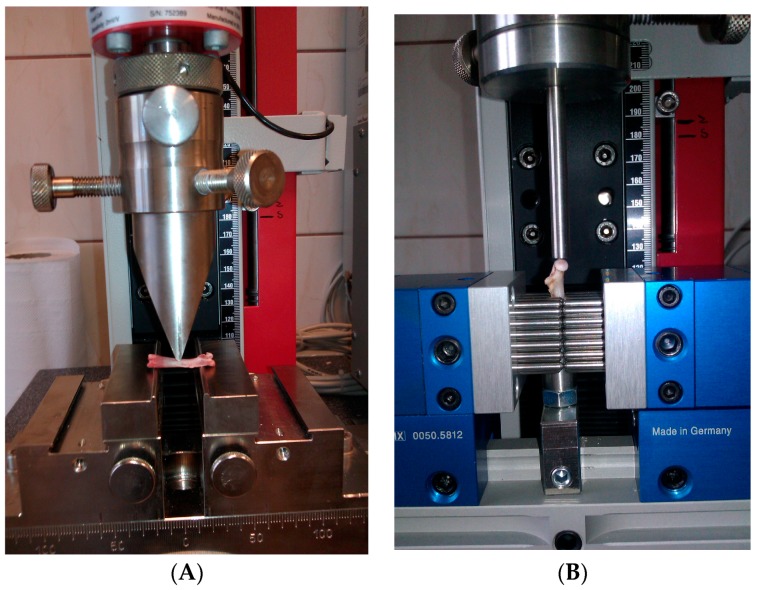
Representation of the biomechanical testing of the femur (the photos originate from our private collection). (**A**) Three-point bending test of the femoral diaphysis; (**B**) Fracture test of the femoral neck.

The femur was subjected to the mechanical study performed using a testing machine (Zwick 2.5; Zwick GmbH & Co. KG, Ulm, Germany) equipped with a load cell (range of forces up to 2.5 N) and a computer for data acquisition and storage (TestXpert II V3.31 software; Zwick GmbH & Co. KG, Ulm, Germany). A three-point bending test (Section 2.5.1) was performed to estimate the biomechanical properties of the femoral diaphysis (Figure 1A) [15]. Next, the proximal end of the broken femur was used for the measurement of the biomechanical properties of the femoral neck in a bending test (Section 2.5.1; Figure 1B) [15]. All of the tests were performed by the same operator. According to the producer, the measurement error of the method is 0.12% of the value recorded.

The load-deformation curves were recorded during the testing (Section 2.5.1 and Section 2.5.2). The yield load and fracture load were determined from the load-deformation curve and computer readings. The yield load is defined as the force causing the first bone damage visible in the load-deformation curve (the force at which the load-deformation curve broke from linearity), whereas the fracture load is the force causing bone fracture. These two forces (yield load and fracture load) describe the bone strength. The yield load (yield strength) reflects the maximum load-bearing capacity under elastic conditions, whereas the fracture load (fracture strength) is the force necessary to cause the bone fracture. Moreover, the bone stiffness was automatically calculated from the linear portion of the load-deformation curve as the ratio of the yield load and the bone deformation at the yield point. The area under the load-deformation curve, reflecting the total energy absorbed by the bone during the test, was measured as work to the bone fracture.

#### 2.5.1. Three Point Bending Test of the Femoral Diaphysis

The femur placed horizontally on two rounded supporting bars, located at a distance of 18 mm, was loaded at the midpoint of the diaphysis in the A–P plane by lowering the third bar (stainless steel pin) at a rate of 2 mm/min, until the bone fracture (Figure 1A).

After finishing the three-point bending test, the vertical (A–P orientation) and horizontal (M–L) internal and external heights at the point of diaphysis fracture were measured. Based on the internal and external heights, geometric properties such as the wall thickness (WT), cross-sectional properties of the M–L axis like cross-sectional area (CSA), and cross-sectional moment of inertia (CSMI) at the point of diaphysis fracture were calculated [15].

The yield load, fracture load, and stiffness describe the “structural” properties of the femur as a whole anatomical unit. To better estimate the biomechanical properties of the femur, the “material” (intrinsic; independent of the tissue size) properties of the bone tissue at the femoral diaphysis were also evaluated. For this purpose, the structural properties of the femoral diaphysis, such as the yield load, fracture load, and stiffness, were normalized for their “geometric” properties, giving the yield stress, fracture stress, and elastic modulus (Young modulus of elasticity), respectively [15]. The yield stress and fracture stress reflect the strength of the bone tissue at the femoral diaphysis, and the values of these parameters were automatically calculated based on the recorded forces and the vertical and horizontal internal and external heights at the point of diaphysis fracture in the three-point bending test [15].

#### 2.5.2. Fracture Test of the Femoral Neck

The proximal end of the broken femur was used for the measurement of the biomechanical properties of the femoral neck (Figure 1B) [15]. The proximal part of the femur was immobilized with special equipment of the Zwick 2.5 testing machine. Next, the bone head was vertically loaded at a displacement rate of 6 mm/min, until the neck fracture [15].

### 2.6. Determination of PC I

After the femur biomechanical testing, slices of the bone tissue collected from the femoral distal epiphysis (trabecular bone region) and diaphysis (cortical bone region) were separated, with the aim of determining the PC I concentration. The bone tissue was crumbled and washed in ice-cold physiological saline (0.9% sodium chloride), to eliminate the remove-available bone marrow. Next, known weight bone slices were homogenized in cold potassium phosphate buffer (50 mM, pH = 7.4) at 4 °C using a high-performance homogenizer (Ultra-Turrax T25; IKA^®^, Staufen, Germany) equipped with a stainless-steel dispersing element (S25N-8G; IKA^®^) to receive an appearing homogenous liquid. The prepared 10% homogenates were centrifuged (MPW-350R centrifugator, Medical Instruments, Warsaw, Poland) at 700× *g* for 20 min at 4 °C, and the aliquots were separated.

The concentration of PC I in the aliquots of the bone tissue was determined with the use of an Enzyme-linked Immunosorbent Assay Kit by Uscn Life Science Inc. (Wuhan, China). The precision of the measurement, expressed as the intra- and inter-assay CV, was <4.8% and 5.2%, respectively.

In order to adjust the bone tissue PC I for the protein concentration, the total protein was assayed in the bone aliquots with the use of a BioMaxima kit (Lublin, Poland).

### 2.7. Statistical Analysis

A one-way analysis of variance (Anova) was used to determine if there were statistically significant (*p* < 0.05) differences among the six experimental groups and then the Duncan’s multiple range post hoc test was conducted for comparisons between individual groups and to determine which two means differed statistically significantly (*p* < 0.05). To discern whether the possible beneficial impact of the AE resulted from an independent action of the extract ingredients and/or their interaction with Cd, a two-way analysis of variance (Anova/Manova, test F) was performed. F values having *p* < 0.05 were considered statistically significant. With the aim of investigating the dependence between the impact of the AE consumption on the biomechanical properties and mineral status of the femur, Spearman rank correlation analysis between the variables describing the bone vulnerability to fracture in the females receiving the extract under the exposure to Cd and previously determined in these animals BMD of the femur [26], was conducted. Correlations were considered statistically significant at *p* < 0.05. All of the statistical calculations were performed using the Statistica package (StatSoft, Tulsa, OK, USA).

## 3. Results

### 3.1. Effect of AE on the Femur Weight in Rats Exposed to Cd

The mean absolute and relative weight of the femur in the control rats ranged from 0.6098 ± 0.014 g and 0.2006 ± 0.005 g/100 g b.w., respectively, after three months, up to 0.7703 ± 0.013 g and 0.1306 ± 0.002 g/100 g b.w., respectively, after 24 months of the experiment. The administration of the AE and Cd alone and together had no impact on the absolute and relative femur weight (Table S6).

### 3.2. Effect of AE on the Geometric Properties of the Femur in Rats Exposed to Cd

The femur length in the control rats ranged from 31.30 ± 0.42 mm after three months to 32.69 ± 0.29 mm after 24 months. The mid-diaphyseal femoral geometric properties such as the M–L and A–P within these animals reached 4.130 ± 0.057 mm and 2.791 ± 0.066 mm, respectively, after three months, and 4.618 ± 0.078 mm and 3.370 ± 0.170 mm, respectively, after 24 months. The administration of the AE and/or Cd for up to 24 months had no impact on the femur length and diameter at the mid-diaphysis (Table S7).

The administration of AE during the whole experiment had no impact on the geometric properties of the femoral diaphysis at the point of its fracture in the three-point bending test (WT, CSA, and CSMI; Table 1). The WT and CSA at the point of diaphysis fracture in the three-point bending test were lower in the animals exposed to the 1 and 5 mg Cd/kg diet for three months (by 14.6% and 15.6%, and 17.7% and 13.2%, respectively), whereas the CSMI was unaffected (Table 1). However, after the longer treatment with Cd, the WT and CSA at the point of diaphysis fracture did not differ compared to the control group, except for the increase (by 12.9%) in the CSA in the Cd_1_ group (Table 1).

### 3.3. Effect of AE on the Biomechanical Properties of the Femoral Neck in Rats Exposed to Cd

**Figure 2:**
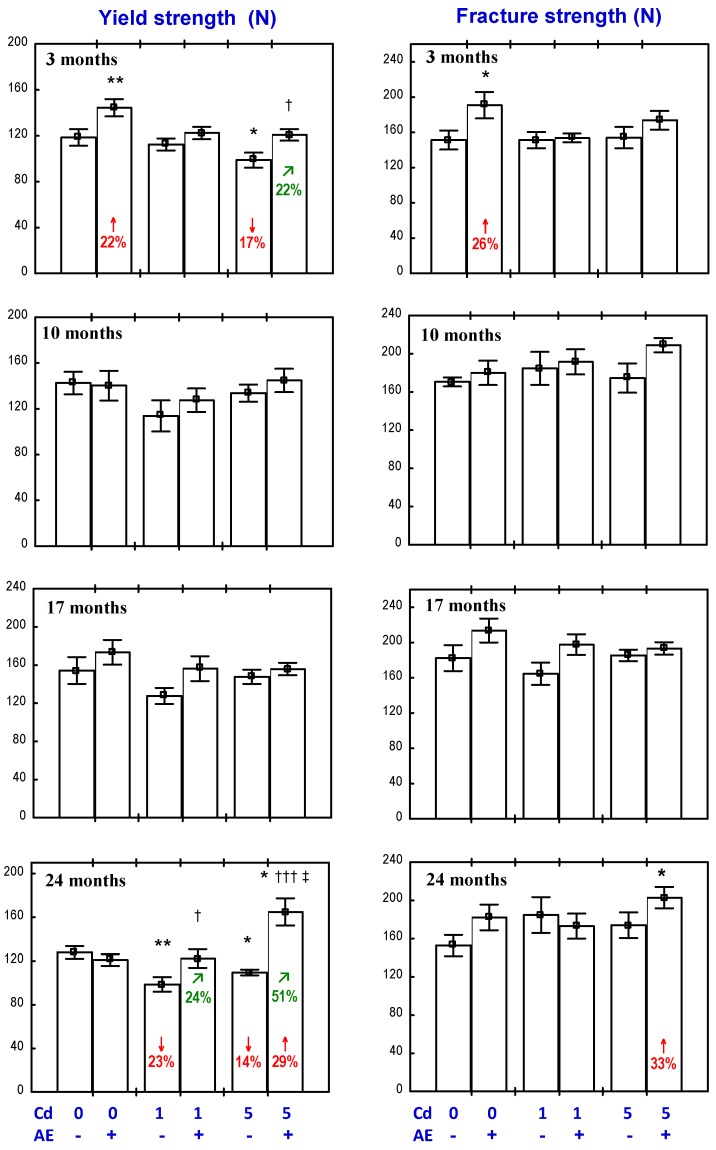
Effect of an extract from the berries of *Aronia melanocarpa* (AE) on the yield and fracture strength of the femoral neck in rats exposed to cadmium (Cd). The rats received 0.1% aqueous AE or not (“+” and “−”, respectively) and Cd in diet at the concentration of 0, 1, and 5 mg/kg. Data are represented as mean ± SE for eight rats, except for seven animals in the AE group, and the groups treated with the 1 and 5 mg Cd/kg diet alone after 24 months. Statistically significant differences (Anova, Duncan’s multiple range test): * *p* < 0.05, ** *p* < 0.01 vs. control group; ^†^ *p* < 0.05, ^†††^ *p* < 0.001 vs. respective group receiving Cd alone; ^‡^ *p* < 0.05 vs. respective group receiving the 1 mg Cd/kg diet. Numerical values in bars indicate percentage change compared to the control group (↓, decrease; ↑, increase) or the respective group receiving Cd alone (↗, increase).

**Figure 3:**
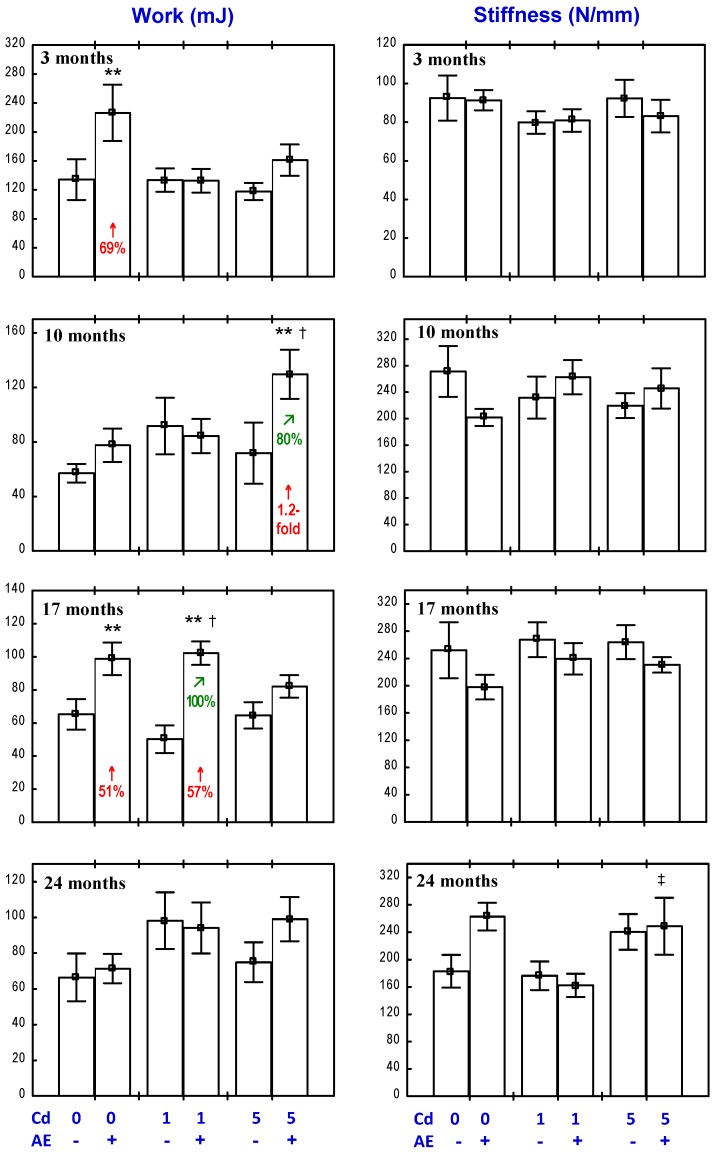
Effect of an extract from the berries of *Aronia melanocarpa* (AE) on the femoral neck stiffness and work to its fracture in rats exposed to cadmium (Cd). The rats received 0.1% aqueous AE or not (“+” and “−”, respectively) and Cd in diet at the concentration of 0, 1, and 5 mg/kg. Data are represented as mean ± SE for eight rats, except for seven animals in the AE group, and the groups treated with the 1 and 5 mg Cd/kg diet alone after 24 months. Statistically significant differences (Anova, Duncan’s multiple range test): ** *p* < 0.01 vs. control group; ^†^ *p* < 0.05 vs. respective group receiving Cd alone; ^‡^ *p* < 0.05 vs. respective group receiving the 1 mg Cd/kg diet. Numerical values in bars indicate percentage change or a factor of change compared to the control group (↑, increase) or the respective group receiving Cd alone (↗, increase).

The administration of the AE alone for three months increased the yield and fracture strength of the femoral neck (Figure 2), as well as the work to the neck fracture (Figure 3), but had no impact on its stiffness (Figure 3). After a longer administration of the extract, all evaluated biomechanical properties of the femoral neck were unchanged compared to the control, except for an increase in the work to the neck fracture after 17 months (Figure 2 and Figure 3).

The exposure to the 1 mg Cd/kg diet for 3, 10, and 17 months had no impact on the biomechanical properties of the femoral neck; however, after 24 months, the yield strength was decreased (Figure 2 and Figure 3). In the animals treated with the 5 mg Cd/kg diet, the only change in the evaluated biomechanical parameters was a decrease in the yield strength after three and 24 months (Figure 2 and Figure 3).

In the animals receiving the AE under the exposure to the 1 mg Cd/kg diet for 17 months, the work to the neck fracture was higher compared to the control group and the group treated with Cd alone (Figure 3). Moreover, the AE completely prevented the 24-month exposure to Cd-induced decrease in the yield strength (Figure 2). The Anova/Manova analysis revealed that the beneficial impact of the AE consumption under the low treatment with Cd on the yield strength of the femoral neck was an effect of the independent action of this extract’s ingredients (F = 4.309, *p* < 0.05) and their interaction with this heavy metal (F = 5.166, *p* < 0.05). The AE administration to the animals treated with 5 mg Cd/kg diet completely prevented the three- and 24-month exposure-induced decrease in the yield strength of the femoral neck (Figure 2), and this effect resulted from the independent action of the extract (F = 10.574, *p* < 0.001) after three months and from its interaction with Cd (F = 17.184, *p* < 0.001) after 24 months. Moreover, the yield strength and fracture strength of the femoral neck in the Cd_5_ + AE group after 24 months were higher than in the control group (Figure 2). The extract administration under the 10-month exposure to the 5 mg Cd/kg diet increased the work to the neck fracture compared to the control group and the Cd_5_ group (Figure 3), as a result of its independent action (F = 4.362, *p* < 0.05).

### 3.4. Effect of AE on the Biomechanical Properties of the Femoral Diaphysis in Rats Exposed to Cd

**Figure 4:**
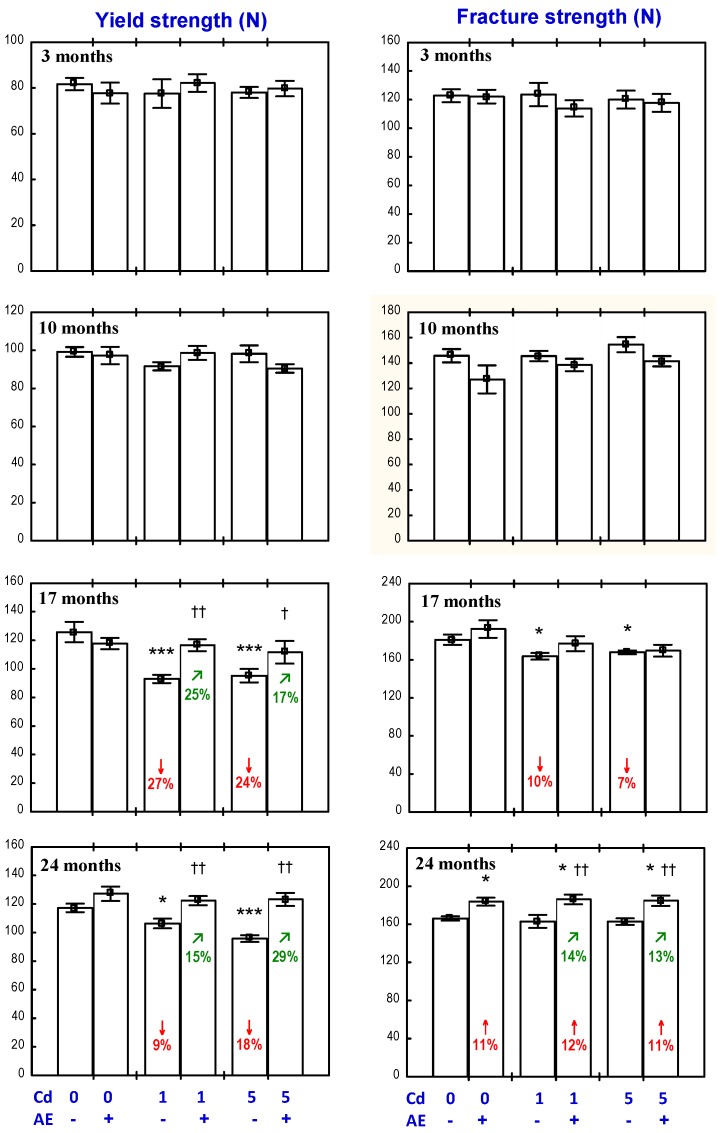
Effect of an extract from the berries of *Aronia melanocarpa* (AE) on the yield and fracture strength of the femoral diaphysis in rats exposed to cadmium (Cd). The rats received 0.1% aqueous AE or not (“+” and “−”, respectively) and Cd in diet at the concentration of 0, 1, and 5 mg/kg. Data are represented as mean ± SE for eight rats, except for seven animals in the AE group, and the groups treated with the 1 and 5 mg Cd/kg diet alone after 24 months. Statistically significant differences (Anova, Duncan’s multiple range test): * *p* < 0.05, *** *p* < 0.001 vs. control group; ^†^ *p* < 0.05, ^††^ *p* < 0.01 vs. respective group receiving Cd alone. Numerical values in bars indicate percentage change compared to the control group (↑, increase; ↓, decrease) or the respective group receiving Cd alone (↗, increase).

**Figure 5:**
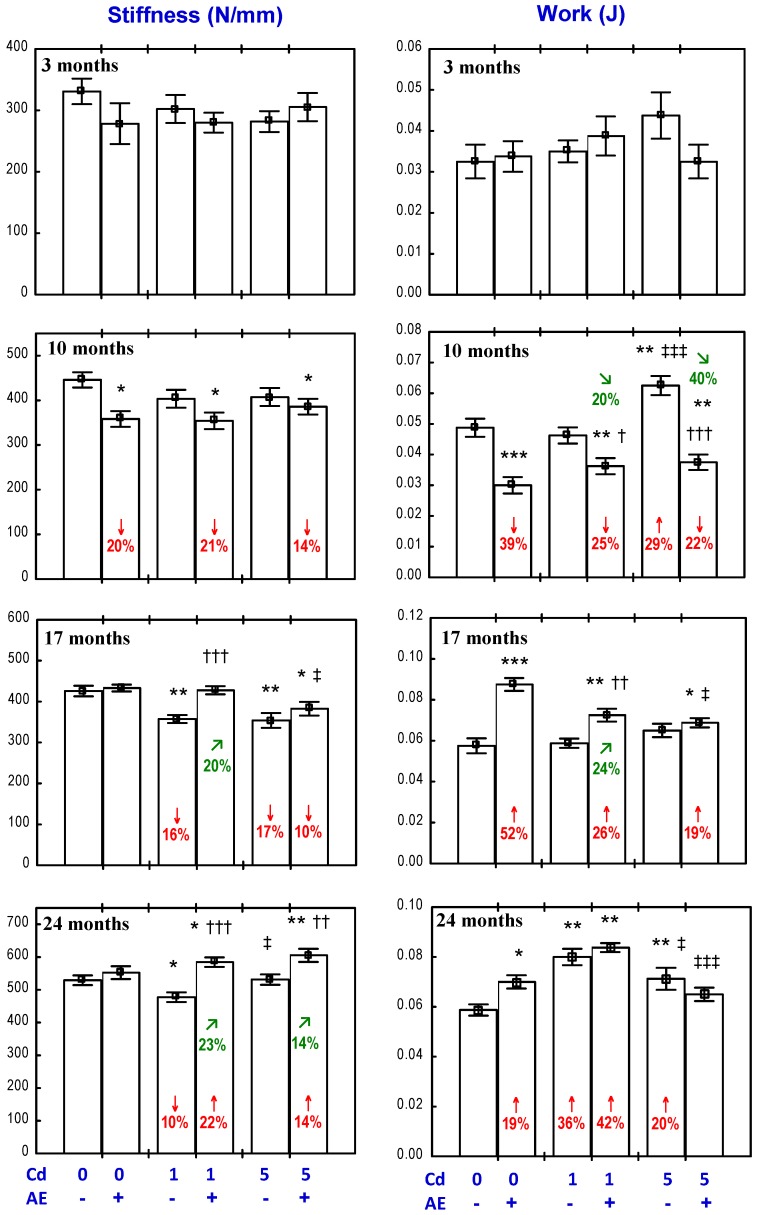
Effect of a polyphenol-rich extract from the berries of *Aronia melanocarpa* (AE) on the femoral diaphysis stiffness and work to its fracture in rats exposed to cadmium (Cd). The rats received 0.1% aqueous AE or not (“+” and “−”, respectively) and Cd in diet at the concentration of 0, 1, and 5 mg/kg. Data are represented as mean ± SE for eight rats, except for seven animals in the AE group, and the groups treated with the 1 and 5 mg Cd/kg diet alone after 24 months. Statistically significant differences (Anova, Duncan’s multiple range test): * *p* < 0.05, ** *p* < 0.01, *** *p* < 0.001 vs. control group; ^†^ *p* < 0.05, ^††^ *p* < 0.01, ^†††^ *p* < 0.001 vs. respective group receiving Cd alone; ^‡^ *p* < 0.05, ^‡‡‡^ *p* < 0.001 vs. respective group receiving the 1 mg Cd/kg diet. Numerical values in bars or above the bars indicate percentage change compared to the control group (↑, increase; ↓, decrease) or the respective group receiving Cd alone (↗, increase; ↘, decrease).

**Figure 6:**
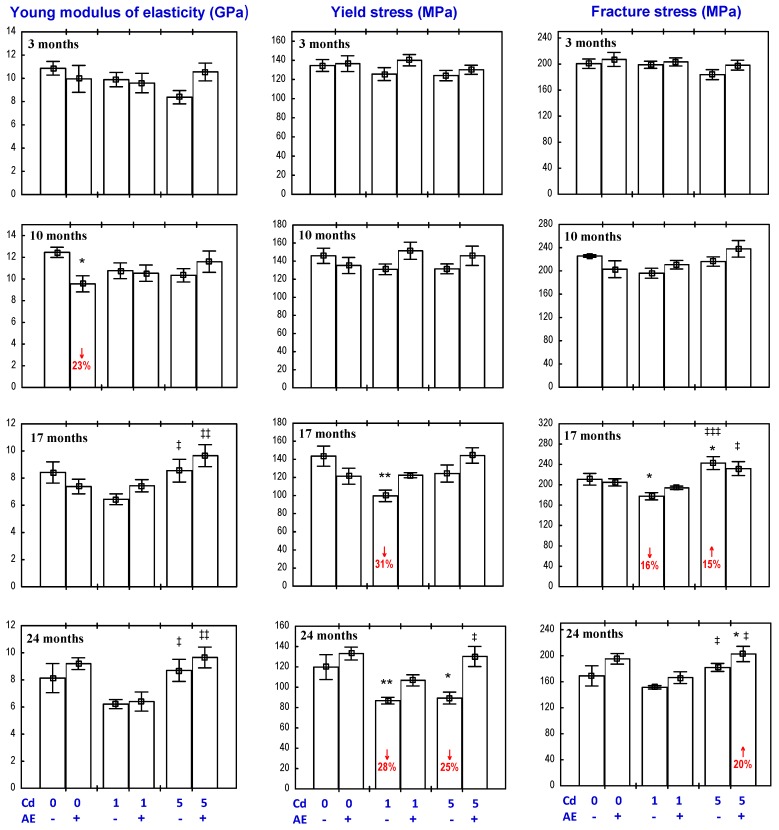
Effect of a polyphenol-rich extract from the berries of *Aronia melanocarpa* (AE) on the “material” biomechanical properties of the femoral diaphysis in rats exposed to cadmium (Cd). The rats received 0.1% aqueous AE or not (“+” and “−”, respectively) and Cd in diet at the concentration of 0, 1, and 5 mg/kg. Data are represented as mean ± SE for eight rats, except for seven animals in the AE group, and the groups treated with the 1 and 5 mg Cd/kg diet alone after 24 months. Statistically significant differences (Anova, Duncan’s multiple range test): * *p* < 0.05, ** *p* < 0.01 vs. control group; ^‡^ *p* < 0.05, ^‡‡^ *p* < 0.01, ^‡‡‡^ *p* < 0.001 vs. respective group receiving the 1 mg Cd/kg diet. Numerical values in bars indicate percentage change compared to the control group (↑, increase; ↓, decrease).

The administration of the AE alone had no impact on the “structural” biomechanical properties (yield strength, fracture strength, stiffness, and work to fracture) of the femoral diaphysis, apart from an increase in the fracture strength after 24 months and a decrease in the stiffness after 10 months, as well as a decrease in the work to fracture after 10 months and its increase after 17 and 24 months (Figure 4 and Figure 5). The only “material” biomechanical property influenced by the consumption of the AE alone was a decrease in the Young modulus of elasticity after 10 months (Figure 6).

The exposure to Cd affected both the “structural” and “material” biomechanical properties of the femoral diaphysis (Figure 4, Figure 5 and Figure 6). In the rats treated with 1 mg Cd/kg diet, the yield strength, yield stress, and stiffness were decreased after 17 and 24 months (Figure 4, Figure 5 and Figure 6). Moreover, after 17 months, the fracture strength and fracture stress were decreased, while after 24 months, the work to the diaphysis fracture was increased (Figure 4, Figure 5 and Figure 6). At the higher exposure to Cd, the yield strength was decreased after 17 and 24 months and the fracture strength and stiffness decreased after 17 months, whereas the work to fracture was increased after 10 and 24 months (Figure 4 and Figure 5). Moreover, the treatment with the 5 mg Cd/kg diet increased the fracture stress after 17 months and decreased the yield stress after 24 months (Figure 6).

The administration of the AE during the exposure to the 1 mg Cd/kg diet completely prevented this heavy metal-induced decrease in the femoral diaphysis yield strength (Figure 4), stiffness (Figure 5), yield stress, and fracture stress (Figure 6). Moreover, the stiffness and fracture strength in the Cd_1_ + AE group after 24 months were increased compared to the control group and Cd_1_ group (Figure 4 and Figure 5). The Anova/Manova analysis revealed that the impact of the AE consumption under the low-level exposure to Cd on the fracture strength of the femoral diaphysis was an effect of the independent action of this extract’s ingredients (F = 8.206, *p* < 0.01) and their interaction with this toxic metal (F = 8.361, *p* < 0.01). Apart from that, the administration of the AE under the 10-month treatment with the 1 mg Cd/kg diet resulted in a decrease in the stiffness of the femoral diaphysis and work to its fracture, and this impact was caused by the interactive action of the extract’s ingredients and Cd (F = 6.194, *p* < 0.05 and F = 7.507, *p* < 0.05, respectively). The administration of the AE under the 17-month exposure to the 1 mg Cd/kg diet increased the work to the femoral diaphysis fracture compared to the control group and the Cd_1_ group, whereas the extract consumption for 24 months had no impact on the Cd-increased value of this parameter. The consumption of the AE under the higher Cd treatment entirely prevented this heavy metal-caused decrease in the yield strength and fracture strength (Figure 4). The beneficial impact on the yield strength resulted from the independent action of the AE after 17 months (F = 6.643, *p* < 0.05) and its interaction with Cd after 24 months (F = 11.43, *p* < 0.01). The 24-month administration of the extract increased the femoral diaphysis fracture strength (Figure 4) and its stiffness compared to the control group and Cd_5_ group (Figure 5), and the diaphysis fracture stress compared to the control group (Figure 6). According to the results of the Anova/Manova analysis, the favourable impact of the AE administration for 24 months on the fracture strength was an effect of its independent action (F = 9.720, *p* < 0.01), whereas the influence on the femoral diaphysis stiffness and yield stress of the bone tissue at the femoral diaphysis resulted from the AE–Cd interaction (F = 5.977, *p* < 0.05 and F = 6.883, *p* < 0.05, respectively). Moreover, this extract administration under the 10-month exposure to the 5 mg Cd/kg diet resulted in a decrease in the work to the diaphysis fracture compared to the control group and Cd_5_ group, increased the value of this biomechanical parameter compared to the control group after 17 months, and completely prevented its increase after 24 months (Figure 5). The Anova/Manova analysis revealed that the impact of the AE administration on the work to the diaphysis fracture after 10 months was an effect of both its independent (F = 14.03, *p* < 0.01) and interactive action with Cd (F = 53.20, *p* < 0.001), whereas after 17 months, this was only a result of the AE–Cd interaction (F = 18.75, *p* < 0.01). Apart from that, the 10-month consumption of the AE under the exposure to the 5 mg Cd/kg diet decreased the stiffness of the femoral diaphysis (Figure 5) as a result of the interaction with Cd (F = 8.219, *p* < 0.01).

### 3.5. Effect of AE on PC I Concentration in the Bone Tissue in Rats Exposed to Cd

**Figure 7:**
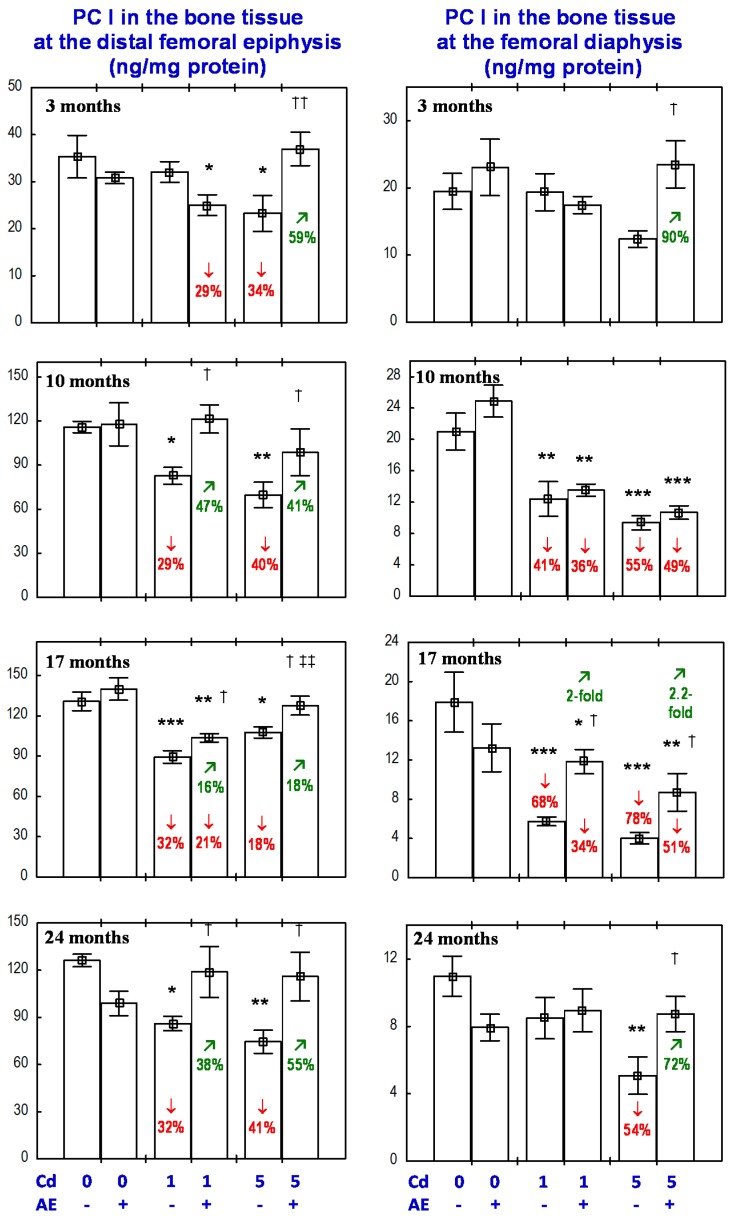
Effect of a polyphenol-rich extract from the berries of *Aronia melanocarpa* (AE) on procollagen I concentration in the bone tissue of rats exposed to cadmium (Cd). The rats received 0.1% aqueous AE or not (“+” and “−”, respectively) and Cd in diet at the concentration of 0, 1, and 5 mg/kg. Data are represented as mean ± SE for eight rats, except for seven animals in the AE group, and the groups treated with the 1 and 5 mg Cd/kg diet alone after 24 months. Statistically significant differences (Anova, Duncan’s multiple range test): * *p* < 0.05, ** *p* < 0.01, *** *p* < 0.001 vs. control group; ^†^ *p* < 0.05, ^††^ *p* < 0.01 vs. respective group receiving Cd alone; ^‡‡^ *p* < 0.01 vs. respective group receiving the 1 mg Cd/kg diet. Numerical values in bars or above the bars indicate percentage change or a factor of change compared to the control group (↓, decrease) or the respective group receiving Cd alone (↗, increase).

The administration of the AE alone for up to 24 months had no impact on the PC I concentration in the bone tissue at the distal epiphysis and diaphysis of the femur (Figure 7).

The exposure to the 1 mg Cd/kg diet for 10, 17, and 24 months resulted in a decrease in the bone tissue concentration of PC I, except for a lack of change in this parameter concentration at the distal femoral diaphysis after 24 months (Figure 7). At the higher exposure to Cd, the concentration of PC I in the bone tissue at the femoral epiphysis was decreased through the whole experiment, while the value of this parameter in the bone tissue at the femoral diaphysis was decreased after the 10th month (Figure 7).

The administration of the AE to the animals fed with the diets containing 1 and 5 mg Cd/kg completely prevented the above-described decrease in the concentration of PC I in the bone tissue at the distal femoral epiphysis, except for a partial protection after 17 months of the exposure to the 1 mg Cd/kg diet (Figure 7). The effect of the AE on the PC I concentration in the bone tissue at the distal femoral epiphysis in the rats exposed to the 1 mg Cd/kg diet was caused by an independent action of the extract (F = 4.621, *p* < 0.05) after 10 months, whereas the impact after 24 months resulted from the interaction of ingredients of the AE with Cd (F = 10.13, *p* < 0.01). Moreover, the tendency of the AE and Cd to interactively impact the PC I concentration after 10 months (F = 3.832, *p* = 0.06) and the extract alone after 17 months (F = 3.680, *p* = 0.06) was noted. The AE consumption under the three-month treatment with the 1 mg Cd/kg diet decreased the PC I concentration in the bone tissue at the distal femoral epiphysis (Figure 7) as a result of an independent impact of the extract (F = 4.246, *p* < 0.05).

The PC I concentration in the bone tissue at the distal femoral epiphysis of the rats exposed to the 1 mg Cd/kg diet for 10–24 months was, on average, 29–32% lower than in the control group, whereas in the animals receiving the AE during the 10- and 24-month exposure to the 1 mg Cd/kg diet, the concentration of this biomarker of collagen biosynthesis did not differ when compared to the control group (and was higher by 47% and 38%, respectively, than in the Cd_1_ group). After 17 months, the value of this parameter was 21% lower than in the control animals (and was 16% higher than in the Cd_1_ group; Figure 7). The exposure to the 5 mg Cd/kg diet for 3–24 months decreased the PC I concentration in the bone tissue at the femoral epiphysis by an average of 18–41% compared to the control group, whereas in the animals receiving the AE under the exposure to Cd, the value of this parameter was within the range of the control group and was an average of 18–59% higher compared to the Cd_5_ group (Figure 7).

The administration of the AE under the 10-month exposure to the 1 and 5 mg Cd/kg diet had no impact on the PC I concentration at the femoral diaphysis; however, in the case of the 17-month co-administration, partial, but clearly evident, protection, resulting from the AE ingredients–Cd interaction (F = 6.771, *p* < 0.05 and F = 4.513, *p* < 0.05, respectively), was noted (Figure 7). The extract consumption for 24 months completely prevented the exposure to the 5 mg Cd/kg diet-induced decrease in PC I concentration at the femoral diaphysis (Figure 7) as a result of the AE–Cd interaction (F = 9.926, *p* < 0.01). Moreover, the three-month administration of the AE to the rats fed with the 5 mg Cd/kg diet increased the PC I concentration at the femoral diaphysis compared to the Cd_5_ group (Figure 7) and the effect resulted from an independent action of the extract’s ingredients (F = 5.603, *p* < 0.05).

The exposure to the 1 mg Cd/kg diet for 10 and 17 months decreased the concentration of PC I in the bone tissue at the femoral diaphysis by 41% and 68%, respectively. In the animals receiving the AE during the 17-month exposure to the 1 mg Cd/kg diet, the concentration of PC I in the bone tissue at the femoral diaphysis was two-fold higher compared to the Cd_1_ group, but it remained lower (by 34%) than in the control group (Figure 7). In the case of the higher level of Cd exposure (5 mg Cd/kg diet), the PC I concentration in the bone tissue at the femoral diaphysis after 17 and 24 months was 78% and 54% lower, respectively, compared to the control group, whereas in the case of the co-administration of Cd and the AE, the concentration was 2.2-fold and 72% higher, respectively, than in the Cd_5_ group. Moreover, after 17 months, the concentration of PC I remained 51% lower than in the control group, while after 24 months, it did not differ compared to the proper values of the control animals.

### 3.6. Dependence Between the Femur BMD and Its Biomechanical Properties in Rats Exposed to Cd

In the animals receiving the AE under the exposure to Cd, positive correlations were noted between the femur BMD and the yield strength (r = 0.525, *p* < 0.001), fracture strength (r = 0.499, *p* < 0.001), stiffness (r = 0.459, *p* < 0.001), and work to the femoral diaphysis fracture (r = 0.445, *p* < 0.001). Moreover, the femur BMD negatively correlated (r = −0.246, *p* < 0.05) with the Young modulus of elasticity of the bone tissue at the femoral diaphysis. A positive correlation was also noted between the femur BMD and the stiffness of the femoral neck (r = 0.260, *p* < 0.05).

## 4. Discussion

The present study is the first that investigated and revealed the protective impact of AE consumption under low-level and moderate chronic exposure to Cd on the bone biomechanical properties and collagen biosynthesis in the bone tissue. Although this study was focused mainly on the possibility of the protective influence of the AE on the femur biomechanical properties under chronic exposure to Cd, it has also provided important data on the effect of low-level chronic exposure to Cd on bone vulnerability to fracture.

The study not only confirmed the previously reported results by us [15,16,17,18] relating to the unfavourable impact of low exposure to this heavy metal on the bone biomechanical properties, but it also provided evidence that the biomechanical properties of the femur may be weakened at a lower exposure than was previously shown and a very low Cd concentration in the blood and urine (0.185–0.324 μg/L and 0.107–0.285 μg/g creatinine, respectively; such concentrations were reached after 17–24 months of the exposure to the 1 mg Cd/kg diet [25]), comparable to the metal concentrations commonly noted in the general population. Weakening of the bone biomechanical properties at such a low Cd concentration in the blood and urine has been reported in the present paper for the first time. The impact of Cd on the biomechanical properties of the femoral neck and diaphysis evaluated in the current study was relatively subtle, but clearly evident taking into account the fact that the unfavourable effect occurred at low-level exposure, resulting in a blood and urinary Cd concentration comparable to this metal’s concentration noted in the general population. The effect of Cd on the femur biomechanical properties in the female rats was less serious than that previously reported by us [15,16,17,18], but the investigated levels of exposure were also lower. The decrease in the yield and fracture strength of the femoral diaphysis after 17 months and in the yield strength of the femoral neck after 24 months provide evidence of decreased bone resistance to fracture, resulting in an enhancement in the risk of fracture due to long-term low-level exposure to this heavy metal. However, it should be clearly underlined that the changes in the biomechanical properties of the femur observed in the present study show that long-term, even very low-level, intoxication with this toxic metal has an unfavourable impact on the skeleton. At both levels of exposure, the impact of Cd on the strength of the femur was clearly evident after only 17 months of intoxication. As was reported in detail in our previous papers, the weakening of the biomechanical properties of the femur due to the treatment with Cd results from the unfavourable impact of this heavy metal on the mineral status and organic matrix (formed mainly by collagen) of the bone [15,16,17,18,33,34]. The proper amount and structure of collagen fibers play an important role in bone formation and strength [29,30,35]. The results of the present study show that the low exposure to Cd (1 mg Cd/kg diet) inhibited collagen biosynthesis in the bone tissue after 10 months. Because the impact of Cd on the “structural” and “material” biomechanical properties of the femoral diaphysis and the femoral neck susceptibility to fracture was previously reported by us [15,16,17,18,33], and the mechanisms leading to an increase in the bone susceptibility to fracture are known and reported, they are not discussed in the present paper. However, the very important finding of the present study regarding the impact of Cd on the femur susceptibility to fracture reveals that this heavy metal may weaken the biomechanical properties of the femoral neck and diaphysis at an exposure lower than has been revealed until now [16,18].

The biomechanical tests performed conclusively revealed that the consumption of the chokeberry extract under long-term low and moderate exposure to Cd improved the femur resistance to fracture. The protective impact of the AE may be explained by the recently reported results by us relating to the beneficial influence of the extract on bone turnover, reflected in an improvement in the femur BMD (Tables S1 and S2) [26] and revealed in the present study’s protection from the Cd-induced inhibition of collagen biosynthesis. The possible mechanism of the extract’s impact on the bone mineral status has been previously reported by us [26] and it consists of an inhibition of Cd-induced bone resorption and the stimulation of the processes of bone formation. It was also revealed by us that the mechanism of osteoprotective action of the AE extract is mediated by its antioxidative properties, as well as the improvement of the oxidative/antioxidative balance in the bone tissue and protection from oxidative stress and oxidative damage to macromolecules in the bone tissue [27].

The results of the Anova/Manova analysis allow us to recognize that the beneficial impact of the AE administration on the biomechanical properties of the femur resulted from both an independent action of the ingredients of the extract and their interaction with Cd, resulting in a lower body burden of this metal and its lower accumulation in the bone tissue [25,26,27]. As a result of the AE ingredients–Cd interaction, the direct and indirect damaging impact of this toxic metal on the skeleton has been weakened. The independent impact of the AE ingredients on the skeleton may be explained mainly by the action of polyphenolic compounds, being the most abundant active components of the aronia berries. Polyphenolic compounds are widely known from their osteoprotective impact related with the improvement of BMD, but the data mainly refers to green tea polyphenols [7,24,36]. In the available literature, apart from our recent reports [25,26,27], there is no data on the impact of the consumption of aronia berries and their products on bone metabolism and bone strength properties. However, our findings in the animals administered the AE alone for 3, 10, 17, and 24 months provide clear evidence for an independent influence of the extract’s ingredients on the skeleton, including the impact on the bone turnover, bone mineral status, oxidative/antioxidative status of the bone tissue, and the bone biomechanical properties [22,23,24]. It seems possible that the main cause of the beneficial impact of the AE under exposure to Cd on the femur biomechanical properties is this extract-caused improvement of the bone mineral status [26]. The positive correlations noted in the current study between the femur BMD and biomechanical properties of the femoral diaphysis (yield and fracture strength, stiffness, work to fracture) in the animals administered with the AE under exposure to Cd confirm the validity of this reasoning. The bone mineral status is one of the main determinants of the bone vulnerability to fracture [28]. Thus, the improvement in the bone mineral status is also related to the improvement in the bone strength properties. Owing to the lack of any literature data on the impact of *A. melanocarpa* on the bone biomechanical properties and the bone susceptibility to fracture, a wider discussion of the results is impossible.

We are aware not only of the achievements, but also of the limitations, of our designed studies on the possible protective impact of AE on the skeleton. At this stage of our investigation, we are unable to explain the exact mechanisms of the beneficial impact of AE on the bone, including the bone susceptibility to fractures, nonetheless our experiment provided unquestionable evidence for this protection. Moreover, because our study was conducted on a female rat model of lifetime environmental exposure, the findings refer to the female skeleton and further study involving an evaluation of AE on the male skeleton is warranted.

## 5. Conclusions

In summary, the present study provides the first evidence that the consumption of AE under chronic exposure to Cd improves the biomechanical properties of the femur, increasing its resistance to fracture at the neck and diaphysis. Fracture is the most serious consequence of the damaging impact of any factor on bone, thus revealing the fact that chokeberry extract enhances the femur resistance to fracture under exposure to Cd is a very important and practically useful finding of our study. This finding, together with our previous results on the bone impact of AE in the experimental model of the lifetime women exposure to Cd [25,26,27], provide strong evidence, allowing us to recognize that the consumption of aronia berries and their products under chronic exposure to this heavy metal seems to be a promising natural strategy for preventing skeletal damage. However, the possible use of the *A. melanocarpa* berries as a prophylactic of bone diseases in humans needs further investigation. It seems necessary to analyse the relationship between the consumption of the aronia berries and the bone status in subjects chronically exposed to Cd, including both males and females.

## Figures and Tables

**Table 1:** Effect of the extract from the berries of *Aronia melanocarpa* (AE) on the geometric properties of the femur at the point of diaphysis fracture in the three-point bending test in rats exposed to cadmium (Cd).

Group	Experiment Duration			
	3 Months	10 Months	17 Months	24 Months
WT (mm)				
Control	0.718 ± 0.047	0.691 ± 0.010	0.786 ± 0.036	0.799 ± 0.018
AE	0.636 ± 0.021	0.693 ± 0.017	0.860 ± 0.034	0.798 ± 0.019
Cd_1_	0.613 ± 0.019 *	0.678 ± 0.018	0.848 ± 0.021	0.899 ± 0.038
Cd_1_ + AE	0.636 ± 0.032 *	0.744 ± 0.023 ^†^	0.756 ± 0.158	0.826 ± 0.051
Cd_5_	0.591 ± 0.020 **	0.721 ± 0.020	0.753 ± 0.023	0.819 ± 0.043
Cd_5_ + AE	0.633 ± 0.015	0.651 ± 0.020 ^†,‡‡^	0.821 ± 0.054	0.869 ± 0.028
CSA (mm^2^)				
Control	7.610 ± 0.386	7.650 ± 0.083	9.114 ± 0.428	9.571 ± 0.247
AE	6.937 ± 0.365	7.642 ± 0.299	10.011 ± 0.478	9.082 ± 0.214
Cd_1_	6.419 ± 0.274 *	7.871 ± 0.235	9.788 ± 0.246	10.810 ± 0.275 *
Cd_1_ + AE	6.902 ± 0.335	8.362 ± 0.440	8.999 ± 0.250	9.926 ± 0.407
Cd_5_	6.602 ± 0.221 *	8.122 ± 0.286	8.485 ± 0.449	9.616 ± 0.457 ^‡^
Cd_5_ + AE	6.775 ± 0.278	7.277 ± 0.396 ^‡^	9.178 ± 0.584	10.039 ± 0.433
CSMI				
Control	3.681 ± 0.208	4.342 ± 0.274	6.338 ± 0.382	8.615 ± 1.682
AE	4.080 ± 0.414	4.980 ± 0.472	7.660 ± 0.612	7.442 ± 0.175
Cd_1_	3.606 ± 0.168	4.882 ± 0.200	6.963 ± 0.609	9.850 ± 0.524
Cd_1_ + AE	3.778 ± 0.205	5.201 ± 0.381	7.061 ± 0.442	9.271 ± 0.200
Cd_5_	4.395 ± 0.390	4.793 ± 0.188	5.239 ± 0.516 ^‡^	8.943 ± 0.726
Cd_5_ + AE	3.721 ± 0.185	4.772 ± 0.356	5.801 ± 0.613	7.762 ± 0.670

Data are represented as mean ± SE for eight rats (except for seven animals in the AE, Cd_1_, and Cd_5_ groups after 24 months). Statistically significant differences (Anova, Duncan’s multiple range test) compared to the control group (* *p* < 0.05, ** *p* < 0.01), respective group receiving Cd alone (^†^ *p* < 0.05), and respective group receiving 1 mg Cd/kg diet alone or with the AE (^‡^ *p* < 0.05, ^‡‡^ *p* < 0.01) are marked. WT, cortical width; CSA, cross-sectional area; CSMI, cross-sectional moment of inertia.

## Supplementary Materials

The following are available online at www.mdpi.com/2072-6643/9/6/543/s1, Table S1: Effect of a polyphenol-rich extract from the berries of *Aronia melanocarpa* (AE) on bone turnover in rats exposed to cadmium (Cd), Table S2: Effect of a polyphenol-rich extract from the berries of *Aronia melanocarpa* (AE) on the femur bone mineral density (BMD) in rats exposed to cadmium (Cd), Table S3: Polyphenolic compounds concentration in the extract from the berries of *Aronia melanocarpa*, Table S4: Cadmium (Cd) intake in particular experimental groups, Table S5: The intake of polyphenolic compounds from the extract from *Aronia melanocarpa* berries (AE) in particular experimental groups, Table S6: Effect of the extract from the berries of *Aronia melanocarpa* (AE) on the absolute and relative femur weight of cadmium (Cd)-exposed rats, Table S7: Effect of the extract from the berries of *Aronia melanocarpa* (AE) on the femur length and diameter at the mid-diaphysis in rats exposed to cadmium (Cd).

## Abbreviations

AE, extract from the berries of *Aronia melanocarpa*; A–P, anterior–posterior; BMD, bone mineral density; Cd, cadmium; CSA, cross-sectional area; CSMI, cross-sectional moment of inertia; CV, coefficient of variation; M–L, medial–lateral; PC I, procollagen I; WT, wall thickness.
