# Supplementary material for: Protective Effect of Chokeberry (Aronia melanocarpa L.) Extract against Cadmium Impact on the Biomechanical Properties of the Femur: A Study in a Rat Model of Low and Moderate Lifetime Women Exposure to This Heavy Metal
Source: Nutrients. 2017 May 25;9(6):543. doi: 10.3390/nu9060543 (PMC5490522; doi:10.3390/nu9060543)
Supplement: Supplementary file 1 [file nutrients-09-00543-s001.zip › nutrients-193776-supplementary.pdf]

Supplementary Materials

Protective Effect of Chokeberry (*Aronia melanocarpa* L.) Extract Against Cadmium Impact on the Biomechanical Properties of the Femur: a Study in a Rat Model of Low and Moderate Lifetime Women Exposure to this Heavy Metal

Małgorzata M. Brzóska*, Alicja Roszczenko, Joanna Rogalska, Małgorzata Gałażyn-Sidorczuk, Magdalena Mężyńska

Department of Toxicology, Medical University of Bialystok, Adama Mickiewicza 2C street, 15-222 Bialystok, Poland

* Correspondence: malgorzata.brzoska@umb.edu.pl; Tel.: +48 85 748 56 04; Fax: +48 85 748 58 34

Table S1. Effect of a polyphenol-rich extract from the berries of *Aronia melanocarpa* (AE) on bone turnover in rats exposed to cadmium (Cd). [1]

Marker of bone turnover	Experiment duration (months)	Exposure to 1 mg Cd/kg diet			Exposure to 5 mg Cd/kg diet		
		Effect of Cd alone	Cd + AE		Effect of Cd alone	Cd + AE	
			Effect of Cd + AE	Effect of AE		Effect of Cd + AE	Effect of AE
**Serum**							
OC	3	↔	↔	↔	↔	↔	↔
	10	↓ 46%	↔	↗ 2.2-fold	↓ 34%	↔	↗ 1.9-fold
	17	↓ 48%	↔	↗ 98%	↓ 45%	↔	↗ 68%
	24	↔	↔	↔	↔	↑ 51%	↗ 76%
CTX	3	↔	↔	↔	↔	↔	↔
	10	↔	↔	↘ 20%	↑ 23%	↔	↘ 15%
	17	↔	↔	↔	↑ 21%	↓ 33%	↘ 45%
	24	↑ 47%	↑ 34%	↔	↑ 2.1-fold	↑ 42%	↘ 32%
**Bone tissue at the distal femoral epiphysis**							
ALP	3	↔	↔	↔	↔	↔	↔
	10	↓ 27%	↓ 28%	↔	↓ 27%	↔	↗ 52%
	17	↓ 36%	↔	↗ 52%	↓ 42%	↓ 27%	↗ 26%
	24	↓ 25%	↑ 23%	↗ 65%	↓ 23%	↑ 45%	↗ 87%
OPG	3	↔	↔	↔	↔	↔	↔
	10	↓ 40%	↔	↔	↓ 54%	↓ 32%	↗ 50%
	17	↓ 51%	↑ 37%	↗ 2.8-fold	↓ 80%	↓ 32%	↗ 3.5-fold
	24	↓ 12%	↑ 75%	↗ 2-fold	↓ 67%	↑ 2.3-fold	↗ 6.9-fold
sRANKL	3	↑ 2.3-fold	↑ 2.2-fold	↔	↑ 2.4-fold	↑ 2.4-fold	↔
	10	↑ 2-fold	↑ 2-fold	↔	↑ 87%	↑ 74	↔
	17	↑ 69%	↔	↘ 56%	↑ 39%	↓ 36%	↘ 54%
	24	↑ 2.5-fold	↔	↘ 70%	↑ 34%	↔	↘ 39%
sRANKL/OPG	3	↑ 2.7-fold	↑ 2.5-fold	↔	↑ 2.5-fold	↑ 2.6-fold	↔
	10	↑ 5-fold	↔	↔	↑ 5.6-fold	↔	↘ 58%
	17	↑ 3.5-fold		↘ 84%	↑ 7.3-fold	↔	↘ 87%
	24	↑ 2.7-fold	↓ 60%	↘ 85%	↑ 4.1-fold	↓ 64%	↘ 91%

[1] Detailed data on the impact of AE on bone turnover in rats exposed to Cd have already been reported [26]. In this table only changes ($p < 0.05$) compared to the control group: a percentage or factor of increase (↑) or decrease (↓), and the respective group that received Cd alone: a percentage of decrease (↘) or increase (↗), are indicated. ↔ and ⟷ without statistically significant ($p > 0.05$) change compared to the control and respective group treated with Cd alone, respectively. OC, osteocalcin; CTX, carboxy-terminal cross-linking telopeptides of type I collagen; ALP, alkaline phosphatase; OPG, osteoprotegerin; sRANKL, soluble receptor activator of nuclear factor-κB ligand; sRANKL/OPG, the ratio of sRANKL and OPG.

Table S2. Effect of a polyphenol-rich extract from the berries of *Aronia melanocarpa* (AE) on the femur bone mineral density (BMD) in rats exposed to cadmium (Cd). [2]

Experiment duration (months)	Exposure to 1 mg Cd/kg diet			Exposure to 5 mg Cd/kg diet		
	Effect of Cd alone	Cd + AE		Effect of Cd alone	Cd + AE	
		Effect of Cd + AE	Effect of AE		Effect of Cd + AE	Effect of AE
3	↔	↑ 11%	↔	↔	↔	↔
10	↓ 18%	↔	↗ 14%	↓ 20%	↓ 12%	↗ 10%
17	↓ 22%	↔	↗ 21%	↓ 19%	↓ 8%	↗ 13%
24	↓ 22%	↔	↗ 26%	↓ 23%	↔	↗ 32%

[2] Detailed data on the impact of the AE on BMD of the femur in rats exposed to Cd have already been reported [26]. In this table only changes ($p < 0.05$) compared to the control group: a percentage of decrease (↓), and the respective group that received Cd alone: a percentage of increase (↗), are indicated. ↔ and ↔ without statistically significant ($p > 0.05$) change compared to the control and respective group treated with Cd alone, respectively.

Table S3. Polyphenolic compounds concentration in the extract from the berries of *Aronia melanocarpa*. [3, 4]

**Compound**	**Concentration (mg/g)**
Total polyphenols	612.40 ± 3.33
Total anthocyanins	202.28 ± 1.28
Total proanthocyanidins	129.87 ± 1.12
Total phenolic acids	110.92 ± 0.89
Total flavonoids	21.94 ± 0.98
Chlorogenic acid	68.32 ± 0.08
Cyanidin 3-O-β-galactoside	80.07 ± 1.05
Cyanidin 3-O-α-arabinoside	33.21 ± 0.01
Cyanidin 3-O-β-glucoside	3.68 ± 0.01

[3] Total polyphenols, total phenolic acids, flavonoids, proantocyanidins and anthocyanins concentrations in the aronia extract by Adamed Consumer Healthcare were determined spectrophotometrically [26]. Polyphenolic profile of the extract and quantification of chlorogenic acid and anthocyanins (cyanidin 3-O-β-galactoside, cyanidin 3-O-α-arabinoside, cyanidin 3-O-β-glucoside) were carried out by Ultra Performance Liquid Chromatography method [26].

Data are represented as mean ± SE (n = 3).

[4] The polyphenolic profile of *A. melanocarpa* berries is well-known [22,23,31,32]. Anthocyanins, proanthocyanidins (oligomeric and polymeric catechins), flavonols (glycoside derivatives of quercetin) as well as hydroxycinnamic acids (chlorogenic and neochlorogenic acids) are present in the extract from the berries of *A. melanocarpa*. The anthocyanin profile of the extract consists almost exclusively of cyanidin glycosides, namely cyanidin 3-arabinoside, cyanidin 3-galactoside, cyanidin 3-glucoside, and cyanidin 3-xyloside (the first two are predominant), and relatively low amounts of pelargonidin 3-arabinoside and pelargonidin 3-galactoside. Flavonols are the minor class of polyphenols in chokeberries. Quercetin, kaempferol and several quercetin mono- and di-glycosides (quercetin 3-galactoside, quercetin 3-glucoside, quercetin 3-rutinoside, quercetin 3-vicianoside, and quercetin 3-robinobioside) are also detected in aronia berries, but in relatively low concentrations. Chokeberry proanthocyanidins consist exclusively of (-)-epicatechin units bonded by a C4 to C8 linkage. The hydroxycinnamic acids are represented by significant amounts of chlorogenic and neochlorogenic acids [23,31,32].

Table S4. Cadmium (Cd) intake in particular experimental groups. [5]

Group	Cd intake (µg/kg. b.w.)			
	**3 months**	**10 months**	**17 months**	**24 months**
Control	4.709 ± 0.067	2.716 ± 0.060	2.582 ± 0.047	2.597 ± 0.051
AE	4.615 ± 0.065	2.935 ± 0.063	2.410 ± 0.046	2.764 ± 0.059
Cd_1	77.50 ± 1.264***	43.85 ± 0.60***	43.41 ± 0.64***	43.22 ± 0.85***
Cd_1 + AE	80.93 ± 1.06***	46.90 ± 0.84***	40.15 ± 0.77***	48.12 ± 0.94***
Cd_5	383.65 ± 4.44***	235.78 ± 2.55***	214.54 ± 2.83***	253.75 ± 8.63***
Cd_5 + AE	385.92 ± 4.91***	239.66 ± 1.43***	208.75 ± 3.04***	252.06 ± 9.29***

[5] Cd intake in the control group and the group receiving 0.1% aqueous extract from the berries of *A. melanocarpa* (AE group) was calculated based on this metal concentration determined by us in the standard diet (0.0584 mg/kg), while this element intake in the groups exposed to Cd was calculated based on its concentration in the feed declared by the manufacturer (1 or 5 mg Cd/kg) [25,26].

Data are represented as mean ± SE for eight rats (except for seven animals in the AE, Cd_1 and Cd_5 groups after 24 months). Statistically significant differences (Anova, Duncan's multiple range test) compared to the control group (*** $p < 0.001$) are marked.

Table S5. The intake of polyphenolic compounds from the extract from *Aronia melanocarpa* berries (AE) in particular experimental groups. [6]

Group	AE intake (mg/kg b.w.)			
	3 months	10 months	17 months	24 months
AE	91.53 ± 1.12	62.29 ± 1.87	53.22 ± 1.14	47.96 ± 1.56
Cd_1 + AE	97.47 ± 1.45	60.92 ± 1.07	55.00 ± 0.76	49.26 ± 1.57
Cd_5 + AE	92.41 ± 1.30	65.14 ± 1.77	56.02 ± 1.15	48.26 ± 2.07

[6] Polyphenols intake was calculated assuming that the AE contained 65.74% of these compounds (manufacturer data). The intake of polyphenols in the control group, Cd_1 group, and Cd_5 group was recognized to be 0 [25,26]. Data are represented as mean ± SE for eight rats (except for seven animals in the AE group after 24 months). There were no statistically significant differences ($p > 0.05$; Anova, Duncan's multiple range test) between the experimental groups.

Table S6. Effect of the extract from the berries of *Aronia melanocarpa* (AE) on the absolute and relative femur weight of cadmium (Cd)-exposed rats.

Group	Experiment duration			
	**3 months**	**10 months**	**17 months**	**24 months**
	**Absolute weight (g)**			
Control	0.6098 ± 0.014	0.6656 ± 0.020	0.7383 ± 0.020	0.7703 ± 0.013
AE	0.6146 ± 0.023	0.6679 ± 0.039	0.7823 ± 0.033	0.8136 ± 0.013
Cd_1	0.5898 ± 0.017	0.6659 ± 0.013	0.7328 ± 0.015	0.7687 ± 0.018
Cd_1 + AE	0.5757 ± 0.014	0.6935 ± 0.023	0.7607 ± 0.016	0.8006 ± 0.018
Cd_5	0.5773 ± 0.016	0.6693 ± 0.014	0.7325 ± 0.033	0.7675 ± 0.016
Cd_5 + AE	0.6047 ± 0.018	0.6847 ± 0.019	0.7635 ± 0.019	0.8074 ± 0.012
	**Relative weight (g/100 g b.w.)**			
Control	0.2006 ± 0.005	0.1541 ± 0.005	0.1459 ± 0.004	0.1306 ± 0.002
AE	0.198 9 ± 0.008	0.1546 ± 0.009	0.1513 ± 0.006	0.1384 ± 0.002
Cd_1	0.1993 ± 0.006	0.1454 ± 0.003	0.1514 ± 0.003	0.1266 ± 0.003
Cd_1 + AE	0.1925 ± 0.005	0.1562 ± 0.006	0.1509 ± 0.003	0.1348 ± 0.003
Cd_5	0.1833 ± 0.005	0.1511 ± 0.003	0.1517 ± 0.008	0.1342 ± 0.003
Cd_5 + AE	0.1914 ± 0.006	0.1634 ± 0.005	0.1536 ± 0.004	0.1360 ± 0.003

Data are represented as mean ± SE for eight rats (except for seven animals in the AE, Cd_1 and Cd_5 groups after 24 months). There were no statistically significant differences ($p > 0.05$; ANOVA, Duncan's multiple range test) between the experimental groups.

Table S7. Effect of the extract from the berries of *Aronia melanocarpa* (AE) on the femur length and diameter at the mid-diaphysis in rats exposed to cadmium (Cd).

Group	Experiment duration			
	**3 months**	**10 months**	**17 months**	**24 months**
	**Femur length (mm)**			
Control	31.30 ± 0.42	32.34 ± 0.48	31.54 ± 0.43	32.69 ± 0.29
AE	31.12 ± 0.29	32.30 ± 0.46	32.17 ± 0.66	33.18 ± 0.40
Cd_1	32.55 ± 1.40	32.96 ± 0.48	31.20 ± 0.30	32.30 ± 0.16
Cd_1 + AE	30.91 ± 0.18	32.62 ± 0.38	32.29 ± 0.24	32.83 ± 0.36
Cd_5	31.55 ± 0.45	33.03 ± 0.33	31.28 ± 0.32	33.40 ± 0.48
Cd_5 + AE	31.04 ± 0.48	31.78 ± 0.41	31.08 ± 0.24	33.69 ± 0.54
	**M – L with (mm)**			
Control	4.130 ± 0.057	4.219 ± 0.052	4.480 ± 0.086	4.618 ± 0.078
AE	4.096 ± 0.111	4.306 ± 0.099	4.565 ± 0.471	4.461 ± 0.066
Cd_1	3.949 ± 0.096	4.383 ± 0.097	4.526 ± 0.053	4.754 ± 0.057
Cd_1 + AE	3.990 ± 0.162	4.313 ± 0.117	4.546 ± 0.082	4.703 ± 0.045
Cd_5	4.151 ± 0.072	4.306 ± 0.085	4.351 ± 0.168	4.566 ± 0.042
Cd_5 + AE	4.063 ± 0.091	4.200 ± 0.135	4.388 ± 0.076	4.545 ± 0.095
	**A – P with (mm)**			
Control	2.791 ± 0.066	2.760 ± 0.084	3.153 ± 0.067	3.370 ± 0.170
AE	2.943 ± 0.079	2.906 ± 0.113	3.309 ± 0.070	3.351 ± 0.018
Cd_1	2.795 ± 0.042	2.946 ± 0.035	3.213 ± 0.091	3.574 ± 0.074
Cd_1 + AE	2.813 ± 0.057	2.983 ± 0.061	3.345 ± 0.059	3.540 ± 0.044
Cd_5	2.900 ± 0.049	2.988 ± 0.032	2.961 ± 0.090	3.521 ± 0.077
Cd_5 + AE	2.758 ± 0.055	2.788 ± 0.073	2.998 ± 0.105‡‡	3.326 ± 0.089

Data are represented as mean ± SE for eight rats (except for seven animals in the AE, Cd_1 and Cd_5 groups after 24 months).

$^{‡‡}p < 0.01$ vs respective Cd_1 + AE group (ANOVA, Duncan's multiple range test).
